# Development of Tissue-Engineered Model of Fibrotic Scarring after Spinal Cord Injury to Study Astrocyte Activation and Neurite Outgrowth In Vitro

**DOI:** 10.1021/acsbiomaterials.4c01100

**Published:** 2024-09-11

**Authors:** Nikolas Ala-Kokko, Inha Baek, Younghye Song

**Affiliations:** Department of Biomedical Engineering, University of Arkansas, Fayetteville, Arkansas 72701, United States

**Keywords:** spinal cord injury, fibrotic scarring, tissue engineering, astrocytes

## Abstract

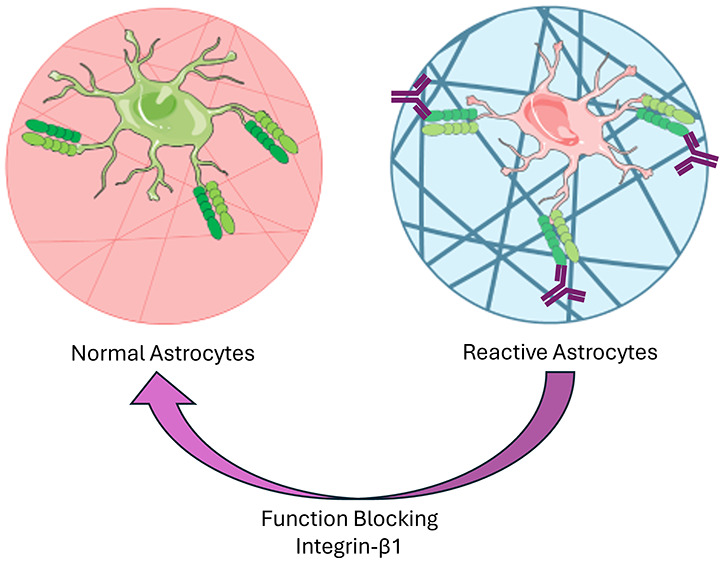

Traumatic spinal cord injuries (SCI) are debilitating injuries affecting twenty-seven million people worldwide and cause functional impairments. Despite decades of research and medical advancements, current treatment options for SCI remain limited, in part due to the complex pathophysiology of spinal cord lesions including cellular transformation and extracellular matrix (ECM) remodeling. Recent studies have increased focus on fibrotic scarring after SCI, and yet much remains unclear about the impact of fibrotic scarring on SCI lesion progression. Here, using collagen and decellularized spinal cord-based composite hydrogels, a three-dimensional (3D) cell culture model mimicking the fibrous core of spinal cord lesions was implemented to investigate its influence on the surrounding astrocytes. To mimic the fibrotic milieu, collagen fibril thickness was tuned using previously established temperature-controlled casting methods. In our platforms, astrocytes in fibro-mimetic hydrogels exhibited increased levels of activation markers such as glial fibrillary acidic protein and N-cadherin. Furthermore, astrocytes in fibro-mimetic hydrogels deposited more fibronectin and laminin, further hinting that astrocytes may also contribute to fibrotic scarring. These markers were decreased when Rho-ROCK and integrin β1 were inhibited via pharmacological inhibitors. Mechanistic analysis of Yes-associated protein reveals that blocking integrin β1 prevents mechanosensing of astrocytes, contributing to altered phenotypes in variable culture conditions. In the presence of these inhibitors, astrocytes increased the secretion of brain-derived neurotrophic factor, and a greater degree of dorsal root ganglia neurite infiltration into the underlying hydrogels was observed. Altogether, this study presents a novel tissue-engineered platform to study fibrotic scarring after SCI and may be a useful platform to advance our understanding of SCI lesion aggravation.

## 1 Introduction

Twenty-seven million people worldwide, including an estimated 18,000 new cases annually in the United States, are affected by spinal cord injury (SCI).^1,2^ SCIs are inflicted by a multitude of causes such as vehicle accidents, sports, trauma, wartime injury, etc., and lead to sensorimotor deficits. Due to the pathophysiology of the injury, the prognosis of many SCIs is an irreversible loss of motor function despite treatment, resulting in a significant decrease in quality of life.^3^ Limitations of the current understanding of pathophysiology stem from the lack of models that are representative of cellular interactions with the extracellular matrix (ECM) leading to the inability to promote regeneration beyond the injury site.

During microenvironment remodeling post-SCI, collagen deposition contributes to a scar environment composed of two distinct regions: fibrous core and glial scarring.^4^ The fibrous core is characterized by increased density and thickness of collagen fibers, resulting in stiffer mechanical properties and variation in how cell binding domains attach to the ECM.^5^ This restructuring of the microenvironment plays a key role in the healing process, in which debris clearing and wound isolation occur.^4^ Consequentially, this results in the inhibition of axonal regeneration past the fibrous core due to the pro-inflammatory and neurotoxic markers stimulated.^6,7^ Glial scarring has been widely investigated in its role post-injury, but cellular interaction with the fibrous core has been largely overlooked until recently.

In this pathological milieu, astrocytes remain present in the surrounding tissue but conform to an altered state. At the boundary of the fibrous core and glial scar, a barrier of astrocytes forms where a phenotypic shift of normal astrocytes to their reactive and scar-forming state, also known as astrogliosis, occurs.^8^ Astrocytes interact with collagen I-rich fibrotic scar via integrin-β1 (ITGB1), and a recent study has shown that this interaction upregulates key scar-forming astrocyte markers like glial fibrillary acidic protein (GFAP) and N-cadherin.^9^ Because of binding domain interactions with the ECM, conformational changes occur in the astrocyte cytoskeleton, resulting in a cascade effect ultimately changing cell shape, adhesion, and behavior.^10^ Of note, adhesion to ECM via integrin-specific binding leads to activation of Rho-associated kinase (ROCK) signaling pathways.^11^ Previous research identified a pharmacological Rho-ROCK inhibitor, Y-27632, as a potential candidate for both astrocytic and neuron regulators.^12,13^ Nevertheless, the therapeutic potential of blocking ITGB1-collagen I interactions and the Rho-ROCK signaling cascade in astrocyte activation in fibrotic scarring remains unclear.

While two-dimensional (2D) in vitro modeling may provide sufficient insights into cellular behavior, many are not a true representation of their native conditions.^5^ With advancements in three-dimensional (3D) cell cultures using various types of scaffolds, 3D environments have been implemented to better mimic physiological conditions in vitro, which the current SCI research largely lacks.^14^ These limitations are particularly prevalent in understanding the fibrotic lesion core, featuring elevated collagen aggregates. Mimicking the fibrous core calls for recapitulating fibro-mimetic ECM composition and organization. The composition can be mimicked by implementing tissue decellularization techniques where cells can be cultured in their host tissue-specific ECM cues.^15^ Fibrotic ECM presentation can be mimicked by tuning collagen fiber thickness via temperature-controlled collagen fibrillogenesis in vitro, where prolonged exposure to colder temperatures results in thicker collagen fibrils assembled in the hydrogels.^5^

In this study, we aim to construct mimics of the fibrous core after SCI by incorporating biologically relevant ECM components to understand astrocyte activation, subsequent fibrotic remodeling, and their consequences on neurite infiltration. To this end, composite hydrogels of decellularized ECM (dECM) from the spinal cord and type I collagen hydrogels were employed. We show that cold-casting of the composite hydrogels modulated phenotypic activation of astrocytes as well as their ECM deposition and brain-derived neurotrophic factor (BDNF) secretion. We identify Rho-ROCK signaling and ITGB1 as primary modulators of astrocyte activation in our testbeds, where pharmacological inhibition of these two signaling pathways resulted in the attenuation of astrocyte activation and increased dorsal root ganglia (DRG) neurite infiltration in vitro.

## 2 Materials and Methods

### 2.1 Astrocyte Cell Culture

Normal human astrocytes (NHAs, Lonza 2565) were cultured in basal media (Lonza 3186 or Sigma M8537) containing the following supplements and growth factors: 2.87% fetal bovine serum (FBS), 0.96% l-Glutamine, 0.0956% GA-1000, 0.0956% ascorbic acid, 0.0956% human epidermal growth factor (hEGF), and 0.24% insulin by volume (all supplements included in Lonza CC-4123). Cells were maintained at 37 °C, 5% CO_2_, until confluency reached 70–90%. Cells between passages 1 and 5 were used in experiments. Media were changed every 48 h.

### 2.2 Collagen I Isolation and Pregel Fabrication

Using established methods, collagen I was isolated from rat tails (VWR RLRBT297).^16^ Briefly, the base of the tail was removed, followed by peeling of the skin. Starting from the tip of the tail, tendons were isolated at each vertebra. Until the completion of all tendon isolations, tendons were stored in 70% ethanol (VWR 89125-172). Isolated tendons were weighed and digested in 0.1% (v/v) acetic acid (Sigma-Aldrich 695092) at 75 mL/g for 3 days at 4 °C. Digested collagen was centrifuged for 90 min at 8800 rpm at 4 °C. Supernatant was frozen for at least 24 h before lyophilization (Labconco FreeZone 4.5) for 4 days (0.01 mbar, −56 °C) and stored at −20 °C. Lyophilized collagen was redigested in 0.1% acetic acid at a concentration of 10 mg/mL, shaken once per day for 4 days, and stored at 4 °C.

### 2.3 Spinal Cord Decellularization

Spinal cord decellularization followed previously established studies.^15,17^ Briefly, porcine spinal cords were purchased from Tissue Source LLC and kept at −80 °C upon arrival. Immediately before decellularization, spinal cords were thawed, dura mater was removed, and decellularization and digestion were performed following a previously established protocol with slight modifications.^15^ Briefly, spinal cord was submerged and agitated in various decellularization baths: sterile deionized water (18 h at 4 °C; 60 rpm), 0.025% trypsin/0.025% EDTA (1 h at 37 °C; 60 rpm; Thermo Fisher Scientific 25300062), 3% Triton X-100 (2 h; 90 rpm; Sigma-Aldrich 93443), 1 M sucrose (1 h; 90 rpm; Sigma-Aldrich S8501), sterile deionized water (1 h; 60 rpm), 4% deoxycholic acid (2 h; 90 rpm, Sigma-Aldrich D6750), 0.1% peracetic acid (Sigma-Aldrich 269336) in 4% ethanol (v/v; 4 h; 90 rpm), 1x phosphate-buffered saline (PBS) (1 h; 90 rpm; VWR), sterile deionized water (twice for 1 h; 90 rpm), and PBS (1 h; 90 rpm). Decellularized SC-ECM were lyophilized (Labconco FreeZone 4.5) for 4 days at 0.01 mbar and −56 °C and kept at −80 °C. Digestion occurred in 0.01 N HCl (Sigma-Aldrich 320331) containing 1 mg/mL pepsin at a concentration of 10 mg/mL, stirred at room temperature (RT) for 3 days, and stored at 4 °C.

### 2.4 Collagen and Spinal Cord Hydrogel Cell Culture

Polydimethylsiloxane (PDMS) microwells were fabricated using soft lithography as previously established.^18^ Briefly, a PDMS Sylgard 184 silicone elastomer kit (Dow Corning) was mixed and fixed on customized silicon wafers, creating cylindrical 3D microwells with a depth of 200 μm and a diameter of 4 or 8 mm. The microwells were then individually placed in a 24 well tissue culture plate (CELLTREAT 229124) and plasma cleaned (Harrick Plasma) followed by the coating of 1% (v/v) polyethylenimine (PEI, Sigma-Aldrich 181978) for 10 min, 0.1% (v/v) glutaraldehyde (Sigma-Aldrich G6257) for 30 min, and washed with deionized water twice.

A combination of the spinal cord and collagen I pregel in a ratio of 1:1 with a final concentration of 5 mg/mL was used in the construction of the 3D environment. 10% media 199 (Sigma-Aldrich M0650) in pregel solution [v/v] was added, followed by neutralization with 1 M NaOH/HCl to adjust the pH to 7.4 and further diluted with 1x PBS. NHAs were seeded at a density of 1 million/mL in 10% media in pregel solution (v/v). Cold- and warm-casting were performed as previously established;^5^ briefly, cold-casting consisting of a prechilling process by placing microwells in well plates on ice was performed, followed by incrementally increasing the temperature while the gels solidified: 4 °C (15 min), RT (10 min), and then 37 °C (10 min). Warm-casting was prewarmed and incubated at 37 °C (30 min). All samples were submerged in AGM for 7 d prior to analysis. Media change occurred every 48 h.

### 2.5 Mechanical Property

The mechanical properties of the hydrogels were measured by compression tests using a rheometer (DHR 2, TA Instruments). Hydrogels for compression test were fabricated in the PDMS mold with a diameter of 8 mm and a height of 3.5 mm, with a volume of 150 μL. Hydrogels were compressed with a load of 250N in between 40 mm diameter stainless-steel parallel plates. All tests were performed at a strain rate of 35 μm/s until failure at room temperature. The data were collected as strain (%) versus strain curve (Pa). Young’s modulus was calculated from the slope of the initial linear region of the stress–strain curve between 5 and 20% of strain.

### 2.6 Gelation Kinetics

The gelation kinetics of complete gels was analyzed without the incorporation of cells and media. Samples were added to a clear 96-well plate on ice and placed in a multiplate reader (SynergyMx) at 37 °C for 45 min. Turbidity was measured at a wavelength of 405 nm. Absorbance readings were normalized to readings at *t* = 0 as previously demonstrated.^19^

### 2.7 Inhibition of 3D Culture

Both function-blocking antibody ITGB1 (Millipore Sigma MAB2253) and pharmacologic inhibitor against Rho-ROCK Y-27632 (Tocris 1254) were added after 72 h of 3D cell culture. ITGB1 antibody was added at 1 μg/mL, and Y-27632 at 10 μM. Treatment was added to all remaining media changes, occurring every 48 h until harvesting.

### 2.8 Hematoxylin and Eosin (H&E) Staining

Tissues were fixed in 3.7% formaldehyde (Sigma-Aldrich 252549) for 18 h, followed by a series of washes with 10% sucrose (Sigma S8501) for 1 day and 30% sucrose for up to 6 days. Following washes, samples were embedded and frozen in Tissue-Tek OCT Compound (VWR 25608-930) at 4 °C for 24 h and then at −80 °C for another 24 h. Cryosections were obtained in the transverse and sagittal planes of the spinal cord, 10 μm thick. After cryosection, samples were warmed at 40 °C for 30 min followed by a series of washes: 1x PBS (two times for 5 min), water (5 min), Harris solution (1 min), water (three times for 1 min), Eosin solution (1 min), 95% ethanol (two times for 1 min), 100% ethanol (three times for 1 min), and xylene (three times for 1 min; VWR MK866816). Coverslips were dried, and DPX mounting media (Sigma 06522) was added to mount a coverslip over samples and set overnight.

### 2.9 Immunofluorescence Staining

After 7 days in culture, the gels were first fixed in 3.7% formaldehyde (1 h at 4 °C), washed in PBS (three times for 5 min), permeabilized in 0.05% Triton X-100 (twice for 7 min), and blocked in 1% bovine serum albumin (BSA, 1 h; Sigma-Aldrich A3294) on a rocker. Primary antibody solutions were incubated on top of the wells at 4 °C overnight. Primary antibodies used included: rabbit anti-GFAP (1:100; Abcam ab16997), rabbit anti-α-smooth muscle actin (α-SMA) (1:100; Abcam ab124964), mouse anti-N-cadherin (1:100; Sigma-Aldrich C3865), rabbit anti-Fibronectin (1:100, Abcam ab2413), rabbit anti-Laminin (1:100, Thermo Fisher PA1-16730), mouse anti-Yes-associated protein (YAP) (1:500, Santa Cruz Biotechnology sc-101199), and mouse anti-neurofilament (1:500, DSHB RT-97). Afterward, wells were rinsed with 0.05% Tween 20 (three times for 5 min; on a rocker; Sigma-Aldrich P9416) and then incubated with secondary antibodies and DAPI (1:2500; Thermo Fisher Scientific D1306) for 1 h. Secondary antibodies: goat antimouse Alexa Fluor 488 (1:500; Thermo Fisher Scientific A11001) and goat antirabbit Alexa Fluor 568 (1:500; Thermo Fisher Scientific A11011). After incubation, samples were washed with 0.05% Tween 20 (twice for 5 min) and PBS (5 min) on a rocker. Samples were submerged and stored in PBS at 4 °C until confocal imaging.

### 2.10 Confocal Microscopy

All samples were imaged using an Olympus IX-83 Automatic Inverted Confocal Microscope using 20x magnification and 2x digital zoom for reflectance imaging. Sample capture settings remained constant across the analyzed sample groups. Volumetric images were taken with less than 5 μm Z-step sizes throughout the gels.

### 2.11 Cytokine Analysis

After 6 days of 3D astrocyte culture, serum-free astrocyte media was added to gels and subsequently harvested after 24 h in culture. Media were then concentrated 20-fold using an Amicon Ultra-4 Centrifugal Filter (3 kDa MWCO; Millipore Sigma; UFC800396). Target-specific Luminex kit (R&D Systems) was used to quantify BDNF, neurotrophin-3 (NT-3), glial-derived neurotrophic factor (GDNF), and β-nerve growth factor (β-NGF). Normalization was performed using dsDNA, which was quantified using Quantifluor dsDNA System (Promega E2670). To isolate dsDNA, aggregates of gels were harvested and subjected to DNeasy Blood and Tissue Kit (Qiagen 69504).

### 2.12 DRG Harvesting and Preparation

All animal work was approved by the Institutional Animal Care and Use Committee (IACUC) at the University of Arkansas. Male Sprague–Dawley rats (Envigo), between 3 and 4 weeks of age, were purchased and housed in the South Camps Animal Facility (SCAF) following IACUC standards and the Animal Welfare Act. Rat DRGs were isolated as previously utilized.^14^

Following euthanasia, the rats were transferred to a sterile hood and placed on an absorbent mat. After the dorsal side of the rat was sprayed with 70% ethanol, electric hair clippers were used to expose the skin from the base of the tail to the top of the neck. Surgical scissors were used to cut the skin along the spine. Afterward, bone cutters were used on the cervical and lumbar regions of the spine, causing total dislocation. Once extracted, a medial dislocation was performed for easier procedures; spinal columns were placed in 1x PBS when not in use. The spinal column was exteriorly trimmed by using surgical scissors to remove excess muscle. Using surgical scissors, the dorsal and anterior sides of the spinal column were cut to expose the spinal cord. The removal of the remaining spinal cord reveals DRGs. Using forceps, DRGs were removed and cleaned using a scalpel. DRGs were stored in hibernate media (Thermo Fisher Scientific; A1247501) until use.

Dissociation was then performed using a previously established method.^14^ DRGs were first placed in a microtube with 0.1% trypsin (Thermo Fisher 15400054) with 1 mg/mL collagenase (Millipore Sigma 10103578001) in 1x PBS (50 min at 37 °C; agitated every 10 min). After incubation, the tubes were centrifuged at 300*g* for 5 min. The supernatant was discarded and replaced with 0.1% trypsin (10 min at 37 °C). Trypsin was then neutralized using 3 times the amount of neurobasal media (Gibco 10888-022) supplemented with 1% Pen/Strep (Thermo Fisher 15-140-122) and 2% B27 (Thermo Fisher A3582801). Samples were centrifuged at 300*g* for 5 min, supernatant was discarded, and DRGs were resuspended in supplemented neurobasal media. Using sterile forceps, a single DRG was placed atop each cell culture gels, and coculture media was pipetted to prevent gels from drying, 10 μL. After 18 h, coculture media were added at typical volumes.

### 2.13 Image Analysis

Intensity analysis for N-Cadherin, GFAP, and α-SMA was performed using the FIJI RGB Measure plugin. Multislice images were compressed using the maximum intensity, thresholding of individual cells, and mean intensity of respective channels was measured. YAP and laminin staining analysis was performed using a customized MATLAB code after a JPEG image of a maximum intensity stacked image was produced in FIJI. This code isolated all channels, constructed a threshold mask of the nuclei, cytoplasm, and whole cells, and output the average intensities. DRG neurite analyses were completed using Sholl analysis of the neuroanatomy plugin in FIJI as previously performed in the laboratory. Volumetric confocal images were compressed via maximum intensity; at 50 μm intervals, measurements were recorded past the edge of the DRG.

### 2.14 Statistical Analysis

Statistical analysis was performed in GraphPad Prism 9 using multiple analysis methods depending on statistical relevance: *t* test and two-way analysis of variance (ANOVA) with Tukey’s posthoc test. Significance was determined at *p* < 0.05. Different letters above each graph represent significant differences; all significant values are provided in Supporting Tables.

## 3 Results

### 3.1 Fiber Thickness Alteration via Temperature Regulation

**Figure 1:**
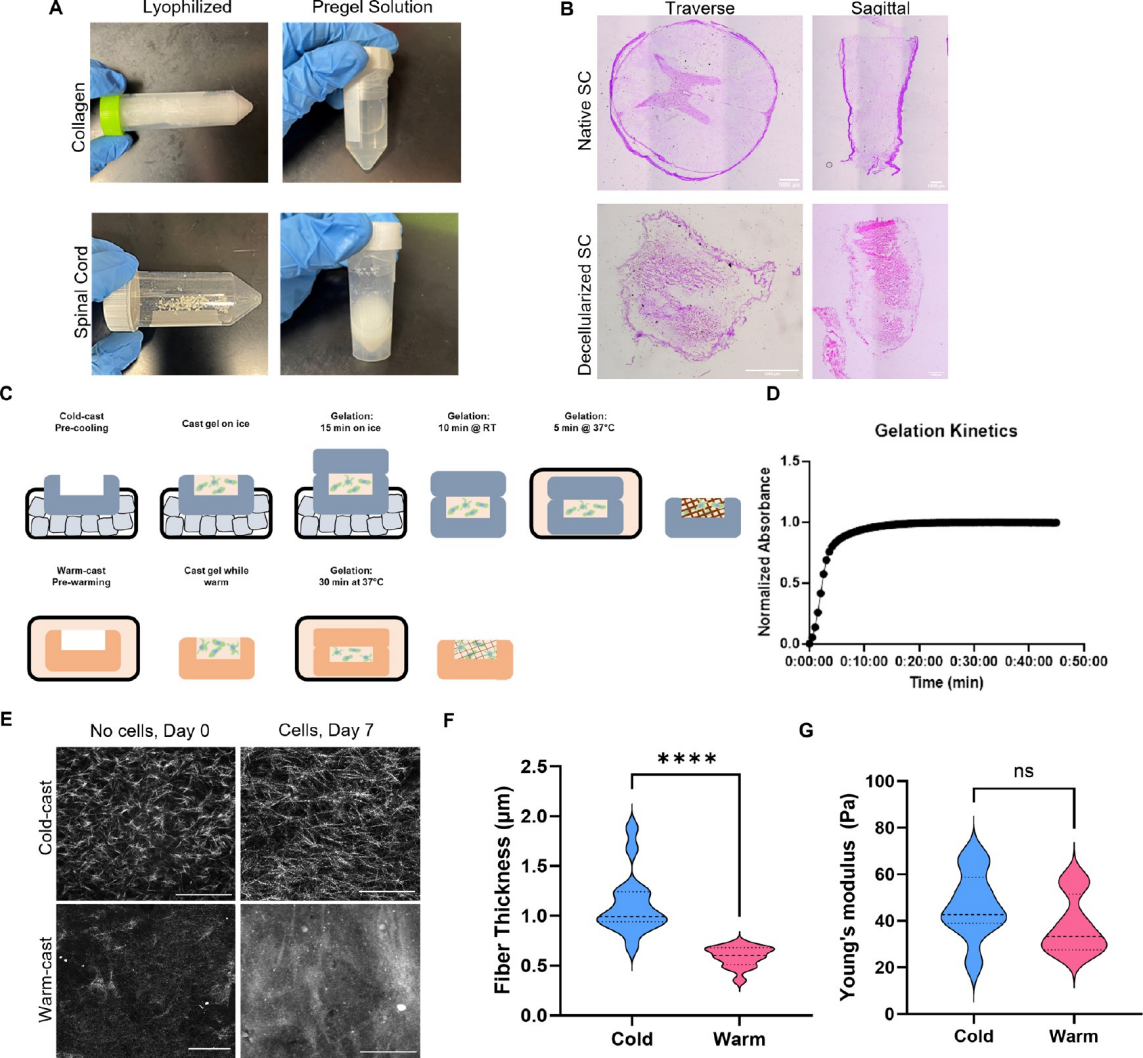
Manufacturing and properties of hydrogel scaffolds. (A) Images of the lyophilized decellularized tissues and digestion for pregel solution. (B) H&E staining of decellularized porcine spinal cord. Scale bar = 1000 μm. (C) Diagram representing variation in fabricating cold- and warm-cast hydrogels for varying fiber thicknesses. (D) Gelation kinetics of composite hydrogels. (E) Confocal reflectance microscopy of ECM on day 0 without cells and on day 7 with cells. Scale bar = 100 μm. (F) Graph showing the collagen fiber thickness after 7 days with cells. (G) Graph representing the mechanical properties of warm- and cold-cast hydrogels. Analysis performed with *t* test, ****p < 0.001 for *n* = 4–9.

Pregel solutions of collagen I and spinal cord ECM were prepared based on established methods of rat tail type I collagen extraction^16^ and spinal cord decellularization^15^ (Figure 1A). Successful decellularization of spinal cords was confirmed via H&E staining of cryosectioned fresh frozen that shows a lack of nuclear staining after decellularization (Figure 1B). After mixing the two pregel solutions, the composite pregel was subjected to cold- and warm-casting as previously established^5^ to mimic fibrotic and healthy spinal cord milieu, respectively (Figure 1C). Thermal self-assembly of ECM fibers leading to hydrogel formation was confirmed via turbidity gelation kinetic measurements at 37 °C (Figure 1D). To characterize ECM fiber thickness, confocal reflectance microscopy (CRM) imaging was performed. As shown in Figure 1E, cold-casting resulted in thicker ECM fibers throughout the composite hydrogels. Importantly, analysis of fiber thickness after 7 days of astrocyte culture shows a continuous elevation in collagen fiber thickness in the presence of cellular activity, with cold-cast gels having a mean of 1.12 μm while warm-casted gels having 0.59 μm thick fibers (*p* < 0.0001) (Figure 1F). Mechanical properties of cold- and warm-cast hydrogels were measured between 5 and 20% of strain. The mean Young’s moduli of cold- and warm-cast hydrogels were 46.70 and 37.99 Pa, respectively (*p* = 0.1996).

### 3.2 Collagen Microarchitecture Regulates Astrocyte Behavior via Rho-ROCK and ITGB1

**Figure 2:**
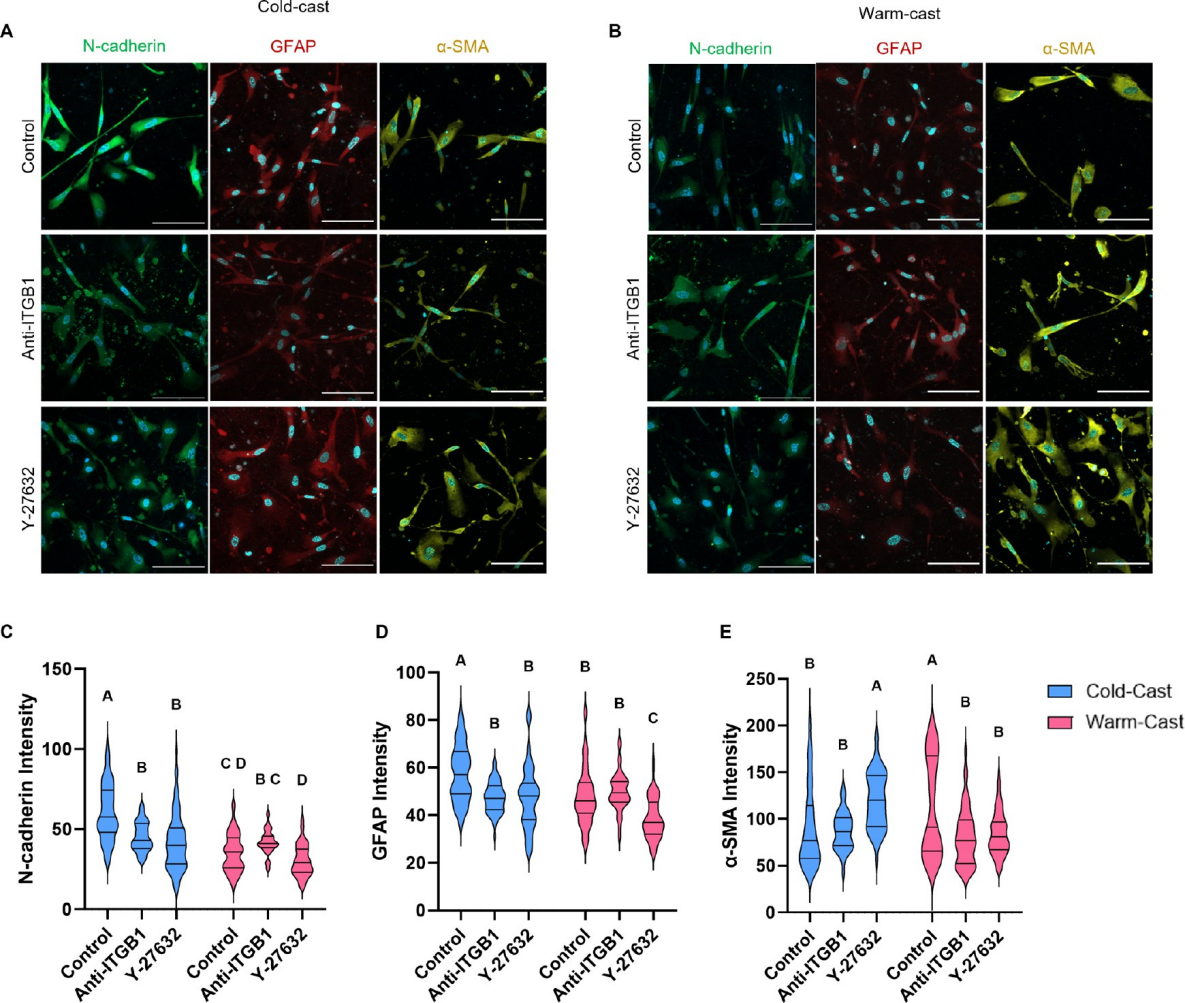
Protein expression of astrocytes in variable scaffold ECM properties and treatments with ITGB1 and Y-27632 immunofluorescence imaging of (A) cold- and (B) warm-cast astrocytes in 3D culture after 7 days of DAPI (blue), N-cadherin (green), GFAP (red), and α-SMA (yellow). Treatments: control, anti-ITGB1, and Y-27632. Scale bar = 100 μm. Mean intensity analysis of (C) N-Cadherin, (D) GFAP, and (E) α-SMA. Two-way ANOVA analysis with posthoc Tukey’s test, *n* = 3–4. Different letters above each graph represent significant differences.

In the cold- and warm-cast hydrogels, normal human astrocytes were embedded at 1 million cells per mL of hydrogels, and evaluation of astrocyte activation was performed via immunofluorescence intensity analysis of N-cadherin, GFAP, and α-smooth muscle actin (α-SMA) (Figure 2A,B). After 7 days of culture, astrocytes embedded in cold-cast hydrogels exhibited increased levels of N-cadherin (*p* < 0.0001) and GFAP (*p* < 0.0001), while α-SMA levels were elevated in warm-cast gels (*p* = 0.0014). Based on recent reports of the collagen I-integrin β1-N-cadherin signaling axis in astrocyte activation,^9^ function-blocking antibodies against integrin β1 (ITGB1) were added to the cultures. In ITGB1-treated samples, astrocytic N-cadherin and GFAP levels decreased in cold-cast hydrogels while they remained unchanged in warm-cast hydrogels. This suggests that the collagen I fiber thickness, not just the presence of collagen I itself, impacts the collagen I-integrin β1-N-cadherin signaling axis in astrocyte activation. Interestingly, ITGB1 blockade did not affect α-SMA levels in cold-cast (*p* = 0.995) while they were reduced in astrocytes cultured in warm-cast hydrogels (*p* = 0.0001) (Figure 2C–E).

On the other hand, Rho-ROCK inhibition using pharmacological inhibitor Y-27632 significantly reduced N-cadherin levels compared to the cold-cast control samples (*p* < 0.0001) while remaining significantly higher than warm-cast control cultures (*p* = 0.0327). When astrocytes were embedded in warm-cast hydrogels and treated with Y-27632, their N-cadherin levels were maintained (*p* = 0.3308). GFAP levels, when treated and compared to their control samples, decreased in both cold- and warm-cast samples (both *p* < 0.0001) equivalent to astrocytes embedded in warm-cast gels (*p* > 0.999). These warm-cast astrocytes further decreased their expression of GFAP when treated with Y-27632 (*p* < 0.0001).

### 3.3 ECM Protein Composition and Expression

**Figure 3:**
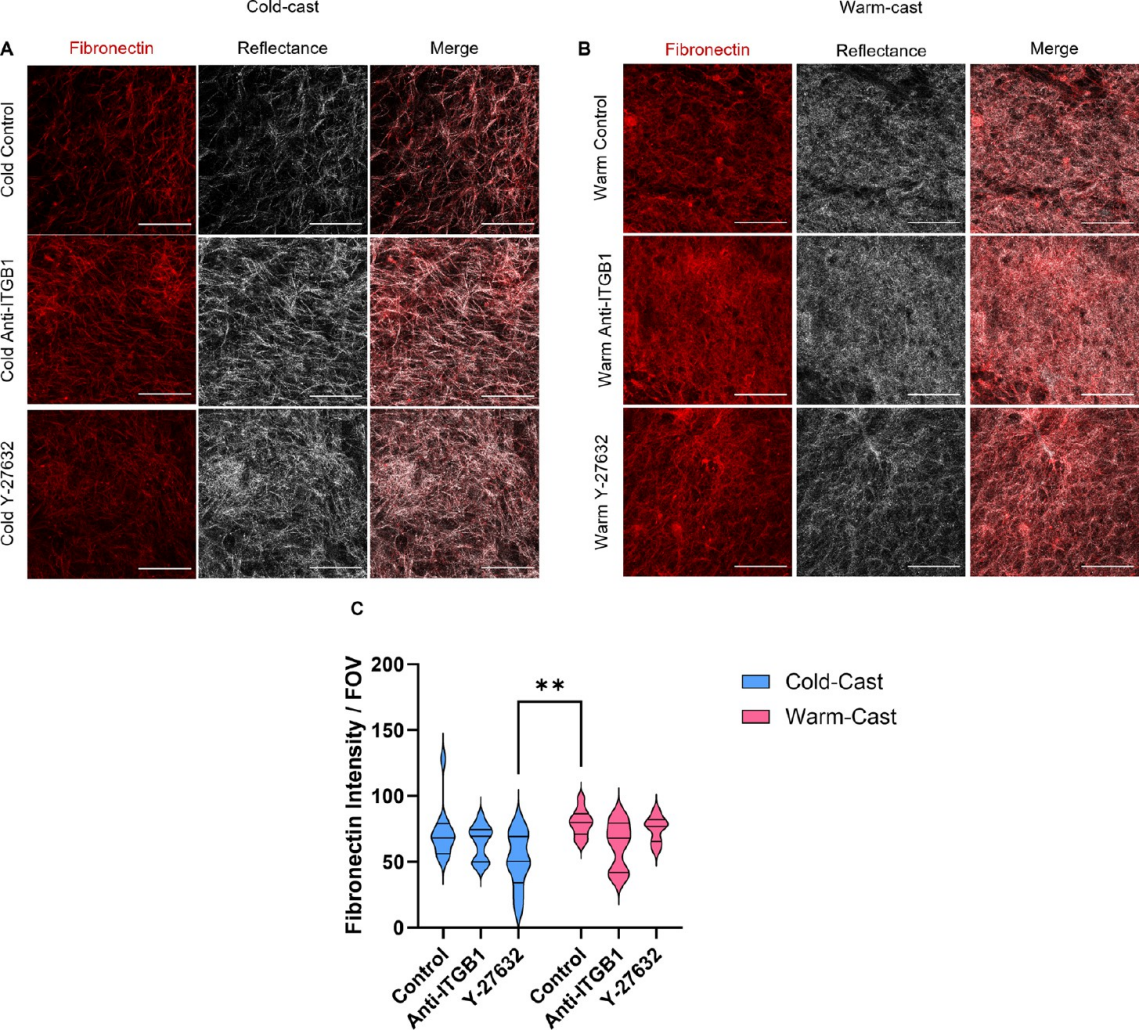
Fibronectin presence in hydrogels. Immunofluorescence and confocal reflectance imaging of (A) cold- and (B) warm-cast ECM proteins, fibronectin (red), and collagen I (white), and imaging of 3D culture containing astrocytes after 7 days with treatment of Y-27632 and ITGB1 blocking. Scale bar = 100 μm. (C) Mean field of view (FOV) intensity analysis of fibronectin. Analysis: two-way ANOVA analysis with posthoc Tukey’s test. **p < 0.01. *n* = 4.

**Figure 4:**
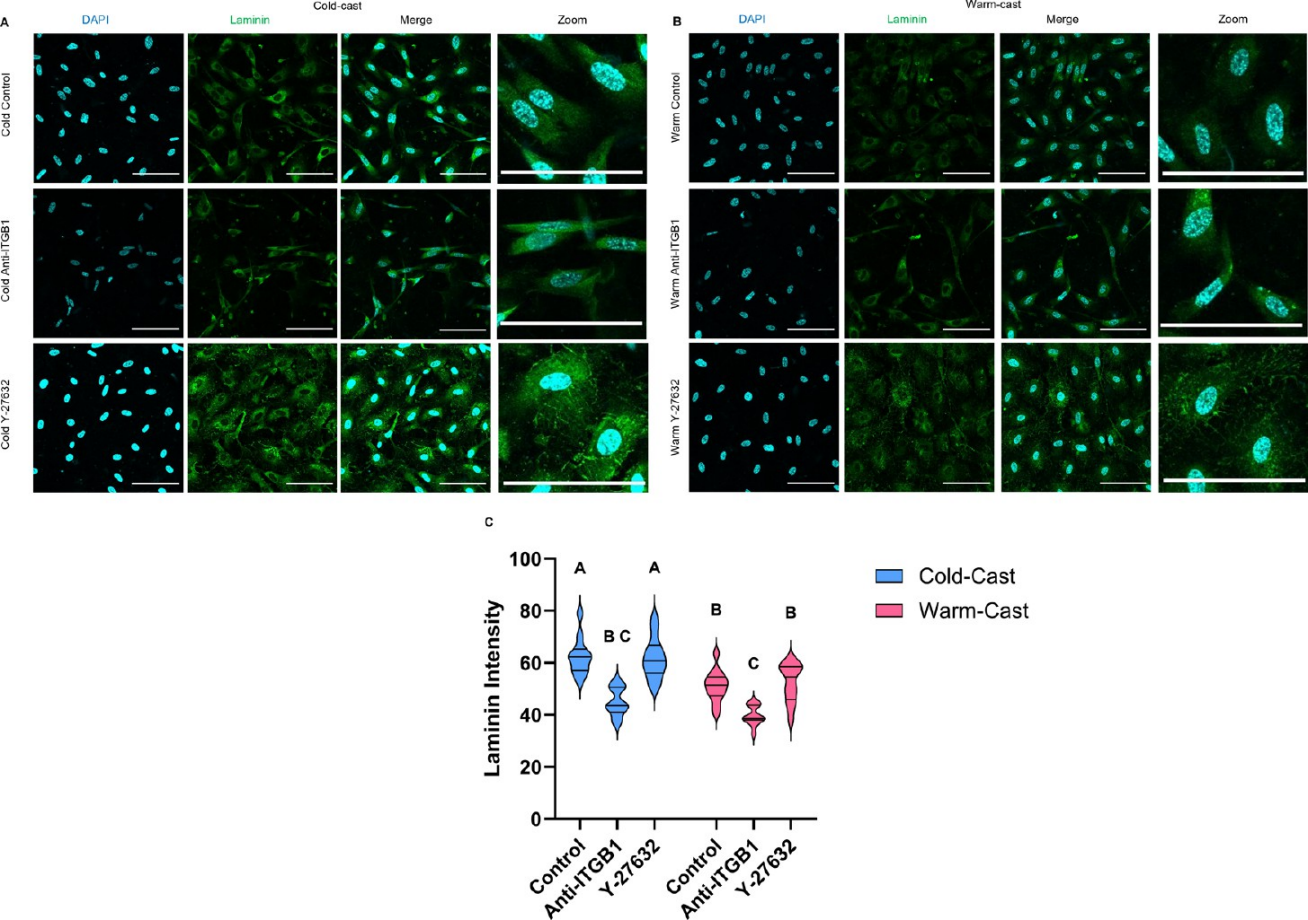
Laminin presence in hydrogel. Immunofluorescence imaging of (A) cold- and (B) warm-cast laminin (green) presence of 3D culture containing astrocytes after 7 days. Treatments: control, anti-ITGB1, and Y-27632. Scale bar = 100 μm. (C) Mean intensity analysis of laminin. Analysis: two-way ANOVA analysis with posthoc Tukey’s test. *n* = 4. Different letters above each graph represent significant differences.

After confirming astrocyte activation in our cold-cast gels, deposition of fibrotic scar ECM components by astrocytes was assessed via immunofluorescence staining of fibronectin and laminin. Fibronectin across most samples remained consistent, yet a significant difference was seen in the cold-cast Y-27632 group against the warm-cast control (*p* = 0.008) (Figure 3A–C). Expression of laminin across groups was imaged to characterize how the astrocyte phenotypic shift regulates the deposition of laminin (Figure 4A–C). When embedded in a fibrous environment, as compared to warm-cast gels, astrocyte cytoplasmic laminin was upregulated (*p* = 0.0008). When astrocytes were treated with ITGB1 function-blocking antibodies, overall levels of laminin in cold-cast samples significantly decreased (*p* < 0.0001) to levels seen in warm-cast control (*p* = 0.2161). When the warm-cast gels were treated with ITGB1 function-blocking antibody, expression of laminin was further reduced compared to their control (*p* = 0.0007). Laminin levels in Rho-ROCK-inhibited astrocytes did not deviate from basal levels in their respective casting methods (*p* > 0.999 and *p* = 0.9965); however, visual inspection revealed altered staining characteristics of cellular laminin, showing a more fibrillar laminin morphology in both cytoplasmic and extracellular space, indicative of new laminin deposition.^20^

### 3.4 YAP Activation Variable in Culture Conditions

**Figure 5:**
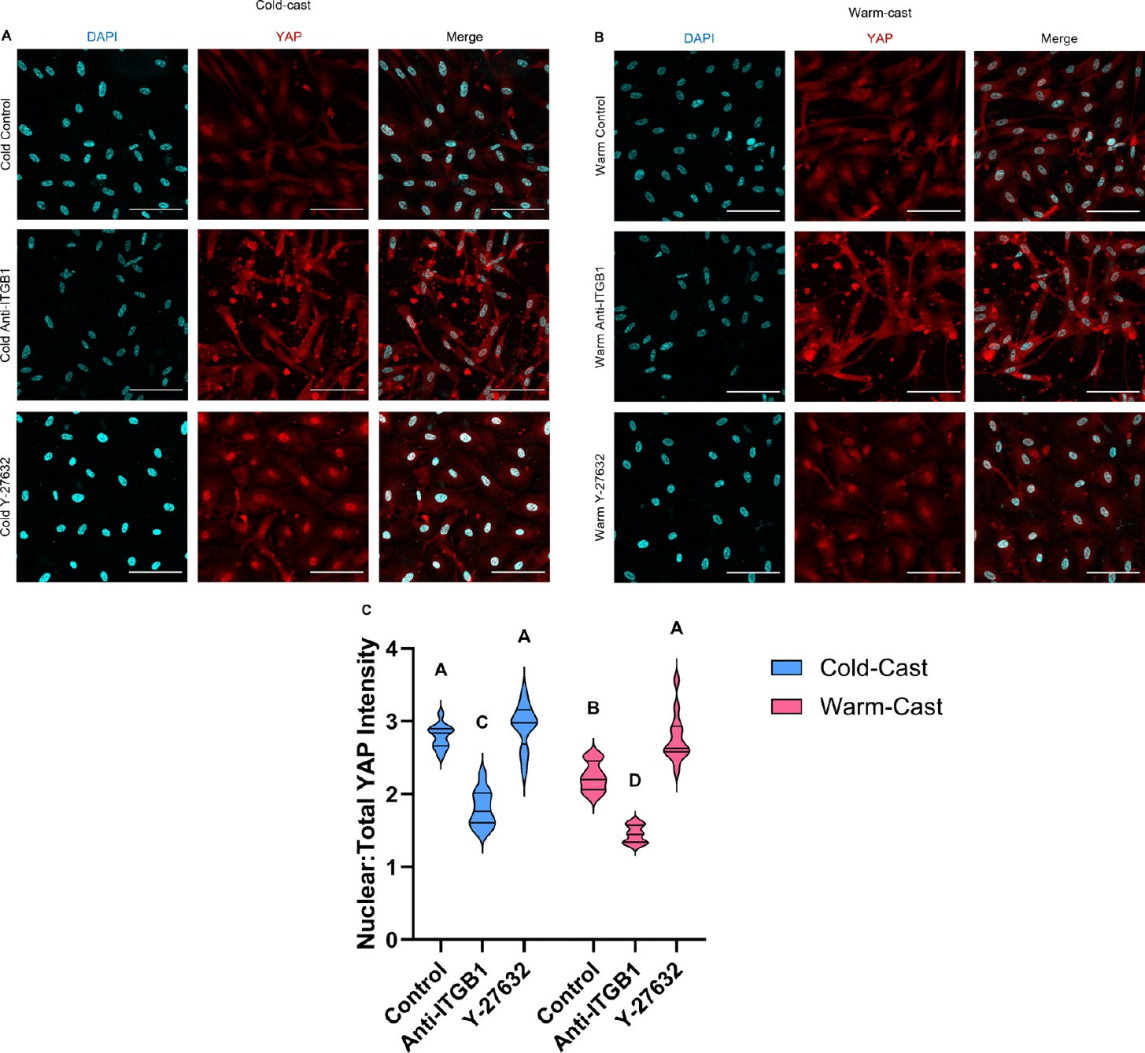
YAP localization in astrocytes. Immunofluorescence imaging of (A) cold- and (B) warm-cast astrocytes in 3D culture after 7 days of DAPI (blue) and YAP (red). Treatments: control, anti-ITGB1, and Y-27632. Scale bar = 100 μm. (C) Nuclear/whole cell staining ratio for YAP. Analysis: two-way ANOVA analysis with posthoc Tukey’s test. *n* = 4. Different letters above each graph represent significant differences.

To understand the mechanism for why astrocytes shift phenotypes based on ECM fiber microarchitectures and how treatment influences astrocyte activation and protein expression, Yes-associated protein (YAP) response was analyzed after 7 days in culture (Figure 5A,B). Nuclear translocation of YAP as a proportion of total expression per cell was determined. On these platforms, astrocytes in cold-cast hydrogels showed elevation in the ratio of nuclear to total cell YAP expression (*p* < 0.0001). By inhibiting the binding of ITGB1, YAP expression was significantly reduced in both cold- and warm-cast samples (both *p* < 0.0001). Treatment with Y-27632, however, kept YAP in astrocyte nuclei in both cold- and warm-cast hydrogels, as no significant difference in cold-cast (*p* = 0.7458) and elevation in warm-cast (*p* < 0.0001) astrocytes were observed when compared to respective controls (Figure 5C).

### 3.5 Cytokine Profile

**Figure 6:**
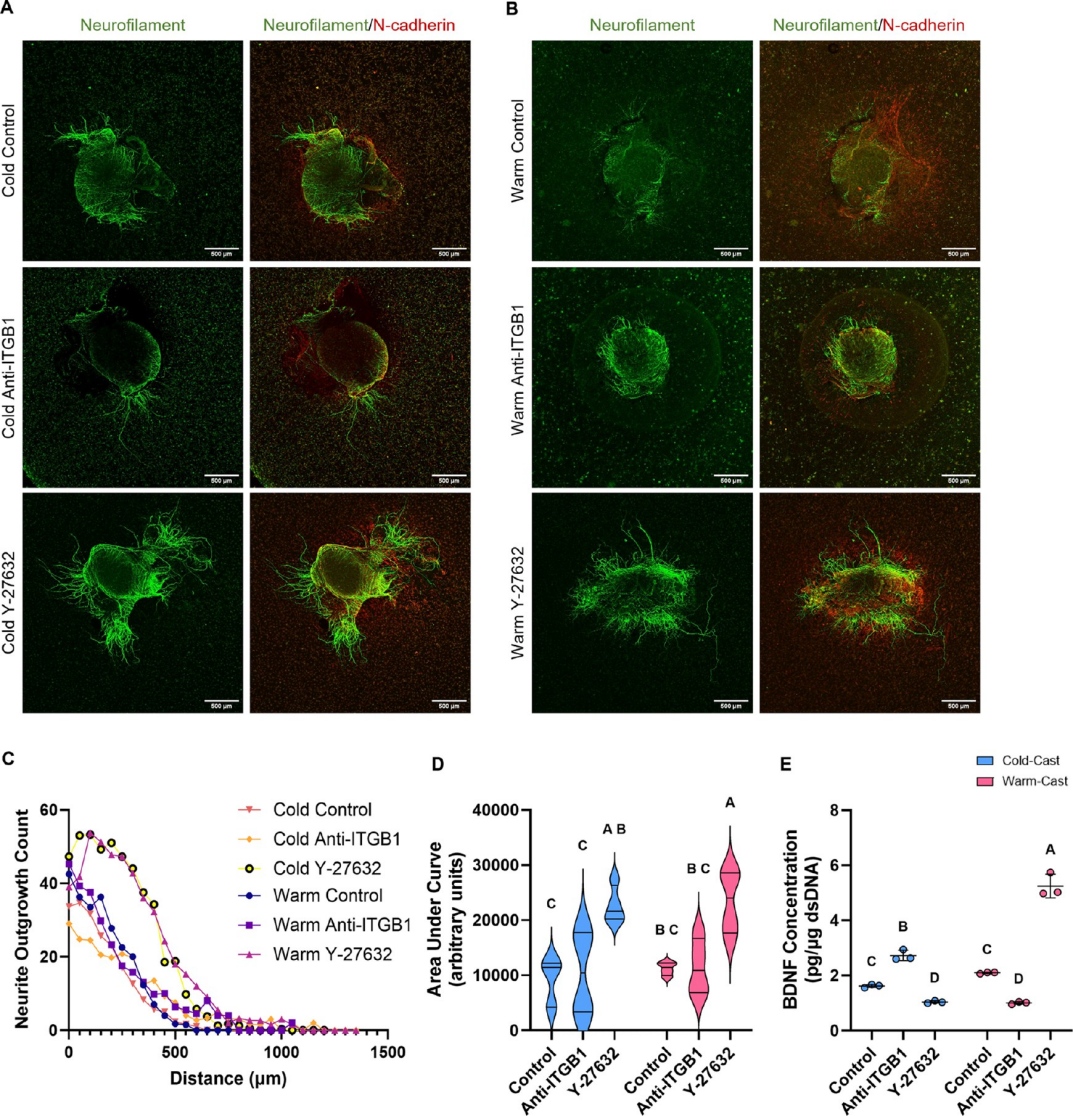
DRG outgrowth models. Immunofluorescence images of neurite outgrowth in (A) cold- and (B) warm-cast astrocyte cultures. Staining of neurofilament (green) and N-cadherin (red). Scale bar = 500 μm. (C) Neurite count at distances past DRG border (μm). (D) The area under the curve produced in (C) neurite outgrowth count vs distance graph. Analysis: two-way ANOVA with posthoc Tukey’s test. *p < 0.05 for *n* = 3–4/group. (E) Graphs representing the results from Luminex analysis of BDNF obtained from astrocytes in respective culture conditions; samples were normalized to dsDNA content. Analysis: two-way ANOVA with posthoc Tukey’s test. *n* = 3. Different letters above each graph represent significant differences.

Astrocyte secretion of neurotrophic factors including brain-derived neurotrophic factor (BDNF), neurotrophin-3 (NT-3), glial-derived neurotrophic factor (GDNF), and β-nerve growth factor (β-NGF) was evaluated across all groups after 7 days in culture. Interestingly, only BDNF was detected from the astrocyte-conditioned medium in all groups. Y-27632-treated warm-cast gels showed a significant increase in BDNF levels from all other groups (Figure 6E). No difference was seen between cold- and warm-cast controls (*p* = 0.0947).

### 3.6 Neurite Outgrowth Elevated with Treatment

To assess scaffold, cell, and treatment dependence on neurite outgrowth, freshly harvested rat DRG were co-cultured atop all platforms. 3D astrocyte cultures were first maintained in regular media for 72 h for acclimation and subjected to ITGB1 or Rho-ROCK inhibition groups for 24 h. Following DRG adherence, treated media was again added to samples for an additional 48 h coculture. Cocultures were then stained against N-cadherin and neurofilament, confocal images were taken, and Sholl analysis was performed to measure neurite extension into the gels (Figure 6A,B). Graphical representation of mean neurite counts at 50 μm intervals is shown in Figure 6C, where the overall analysis was measured by the area under the plotted neurite length versus count curve (Figure 6D). No significant difference was seen in control warm- and cold-cast gels (*p* = 0.9958) while both ITGB1-blocked cold- and warm-cast had no elevated DRG neurite extension (*p* = 0.9995 and *p* > 0.9999). Both Rho-ROCK inhibited gels had the highest overall outgrowth as compared to their respective control casting method (*p* = 0.0308 and *p* = 0.0349) (Figure 6D).

## 4 Discussion

Traumatic SCIs often result in irreversible loss of sensation and motor function. This is attributed to the pathophysiological reaction to maintain homeostasis via repair and injury site isolation. In efforts to promote axonal regeneration, research has previously investigated glial scarring after SCI.^4^ Despite a deeper understanding of glial scars, functional axonal regeneration beyond the lesion sites has seen limited success. To better understand SCI lesions beyond the glial scar, we developed a 3D culture platform that allows for the investigation of astrocyte-ECM interactions at the lesion core, which resembles that of a secondary phase SCI. With our hydrogels comprising type I collagen and decellularized spinal cord, we can model the region beyond the glial scar, which is composed of a high density of astrocytes that interact with the fibrous core at the lesion site.^4^ Casting variance in our collagen-based hydrogels allowed us to tune the microarchitecture of the ECM network and study astrocyte activation in our in vitro model of the SCI lesion fibrotic core. Furthermore, our model also allows the incorporation of neuronal coculture to assess the effect of preventing astrocyte activation on the enhancement of axonal regeneration into a neurotoxic environment.

To first characterize our model, H&E staining of both native and decellularized porcine spinal cords was performed to determine the effectiveness of decellularization. Qualitative evaluation of H&E staining shows slight dysregulation of gray matter structures observed in the transversely sectioned spinal cord and cellular removal, which we have confirmed using DNA analysis following established procedures.^17^ The apparent decrease in structural integrity may stem from the fact that the dura mater was removed from the fresh spinal cord before decellularization.

Composite hydrogels were created by mixing equal parts of collagen I and spinal cord ECM pregel solutions. Our results show successful application of cold- and warm-casting in our composite hydrogels, where elevated fiber diameters were observed in cold-cast gels determined using CRM as previously demonstrated.^5,21−23^ To understand the longevity of these architectures over the culture period, scaffolds containing astrocytes were analyzed on day 7, which signified the maintenance of the ECM in a continued fibrous state. Importantly, our results show that despite the same ECM composition, differences in fiber thickness can greatly affect embedded astrocyte behavior. These results follow the previously established research to investigate fiber thickness on cell behaviors, where fiber organization and its alteration on hydrogel characteristics have been shown to phenotypically change cells.^5^

Interestingly, our mechanical property measurements via compression testing showed slightly higher Young’s modulus in cold-cast hydrogels compared to warm-cast controls. This may be due to the absence of oscillations during compression testing. Previous studies have shown that applying shear stress to the cold- and warm-cast gels aligns ECM fibers, potentiating differences in hydrogel stiffness.^5^ Nevertheless, our studies show distinct differences in fiber thicknesses, where thicker fibers present in cold-cast hydrogels increase astrocyte reactivation. Measuring local stiffnesses in the future would further elucidate mechanosensing-mediated astrocyte activation, including differences in YAP nuclear localization, as discussed below.

Following scaffold characterization, an immunofluorescence intensity analysis of key proteins associated with astrocyte activation was performed. GFAP is an established glial cell cytoskeletal protein that is upregulated in individuals suffering from SCIs.^24,25^ GFAP has long been used as a distinguishing marker for neurological abnormalities including Alzheimer’s disease, brain trauma, amyotrophic lateral sclerosis (ALS), and SCIs.^25−27^ Another cytoskeletal protein, α-SMA, has been identified as a potential astrocyte reactivity maker, as it is elevated when mechanical properties shift in fibrotic scarring due to increased contractility of cells on the ECM.^28,29^ In addition, by regulating activation through adherence to the ECM, the plasticity and prevention of astrocyte activation has been theorized.^9,30^ We also saw increased N-cadherin levels, which aligns with a previous study that identified N-cadherin as a marker for the activation of astrocytes when they bind to collagen via integrin-binding domains.^9^ Consequently, blocking ITGB1 prevents the transition of normal astrocytes to a reactive scar-forming state,^9,31^ which we also observed in our in vitro models.

Cellular response to the varied collagen fiber thickness may result in altered mechanosensing in astrocytes, including Rho-ROCK activation.^24,28^ The Rho-ROCK signaling pathway is involved in glial and neuronal migration, development, and adhesion.^11^ ROCK is the downstream effector of RhoA, mediated by integrins. Therapeutic targeting of ROCK has been implemented in a range of applications like cancer, central nervous system (CNS) disorders, and cardiovascular disease.^32^ SCIs have been shown to activate RhoA in both nerve and glial cells but with pharmacologic inhibition using Y-27632 regenerative properties of neurons, and astrocyte phenotype shifts occur.^33,34^ Like the Rho-ROCK pathway, ITGB1 is responsible for the cell-ECM interaction. Contributing to major adhesive capabilities, specifically to the highly abundant collagen I, ITGB1 is responsible for cell support and structure resulting in a cascade influencing cell function.^10^ Understanding the role of ITGB1 on astrocytic wound repair after SCIs may provide therapeutic options, as previously established research demonstrated collagen I-ITGB1-dependent phenotypic shift of astrocytes via N-cadherin in vivo.^9^ Here, similar approaches in astrocyte treatment were established for the investigation of Rho-ROCK and ITGB1 pathways. Interestingly, inhibiting Rho-ROCK and ITGB1 in cold-cast gels had a greater effect on reducing astrocytic GFAP and N-cadherin levels than in warm-cast gels.

Fibrotic lesions comprise collagens, fibronectin, and laminin.^8^ Following SCI, elevated deposition of these ECM proteins contributes to the altered cell behavior. Excessive fibronectin deposition and prevalence in the CNS could contribute to the pro-inflammatory and neurotoxic environment, while others report the pro-regenerative need for both fibronectin and laminin.^11,35−38^ Fibronectin, a component in both native and upregulated forms in the injured spinal cord, serves as a mediator of cell adhesion and migration. Integrin α5β1 is responsible for the binding of cells to fibronectin, where subsequent activation of RhoA and downstream ROCK results in the contractility and cytoskeletal remodeling of cells.^39^ While fibronectin analysis revealed unchanging intensity across most sample groups in this study, the presence of fibronectin was in accordance with both thin and thick fibers from warm- and cold-casting methods, respectively. This is consistent with previous studies where fibronectin aggregated with collagen fiber binding sites and resembles the morphology of collagen.^40^ While fibronectin is deposited throughout, laminin has been shown to form around the periphery of the lesion site following SCI.^8,41,42^ This is indicative of the duality of the astrocytic reactivity. Our results show fibrillar laminin in the cytoplasmic and extracellular space of astrocytes treated with Y-27632 in both cold- and warm-cast hydrogels, while astrocytes in control and ITGB1 neutralization groups showed diffuse cytoplasmic laminin staining. Interestingly, others have reported limited success in showing any upregulation of collagen, fibronectin, or laminin production by glial cells cultured in collagen gels with ROCK inhibition via Y-27632.^43^ Further, we also show that while Y-27632 treatment of astrocytes did not alter laminin levels in both cold- and warm-cast hydrogels, ITGB1 neutralization significantly reduced laminin levels. Our data add to the demonstrated importance of ITGB1-laminin interaction roles in astrocyte adhesion and migration especially in the context of wound healing.^44,45^

Nuclear translocation of YAP was assessed to investigate the underlying mechanism causing the astrocyte phenotypic shift in variable hydrogel architectures and treatments. YAP is a transcription factor that contributes to cell growth, and its activation results in translocation to the nucleus. Upstream regulators of YAP range from growth factors, mechanical cues, integrin binding, and cell–cell communication.^46,47^ By controlling both the influences of ROCK and ITGB1 signaling, YAP activation may be directly influenced in our cultured astrocytes. Here, we saw a fiber thickness-dependent nuclear localization of astrocytic YAP, as astrocytes in cold-cast gels had a significantly higher fraction of nuclear YAP compared to those in warm-cast gels. Interestingly, while the ITGB1 blockade significantly reduced YAP nuclear localization, the Y-27632 treatment did not alter YAP activation. Our data add to currently conflicting information existing on Y-27632 treatment and astrocyte activation and require further investigation to unravel mechanisms of Rho-ROCK inhibition on astrocyte behavior as a function of ECM microarchitecture.

Connecting the central and peripheral nervous systems, DRGs are widely used in the modeling and investigation of neuronal outgrowth.^48−51^ Sholl analysis, where neurite measurements began immediately after the boundary of the DRG, was used to quantify neurite outgrowth laterally in the gel. Y-27632-treated gels had the highest overall neurite outgrowth beyond the DRG border, with no distinction depending on scaffold characteristics. Previous work via inhibiting the ROCK pathway shows contradictory outcomes when exploring 2D vs in vivo studies. When cultured atop, a monolayer of astrocytes Y-27632 showed benefits to neurite myelination but no significant outgrowth.^52^ Conversely, ROCK inhibitors show overall elevated levels of axonal regeneration in other 2D cultures and in vivo settings.^53−56^ Here, Y-27632 was added after 72 h and continued treatment following DRG seeding. Subsequent analysis of the effect Y-27632 has on astrocytes alone to regulate neurite infiltration in an injury model will need to be investigated. Using our models, despite not observing infiltration differences as a result of casting influences on astrocytes, with the treatment of our coculture platforms, there is an observed responsive behavior of innervation of the hydrogels with the presence of astrocytes.

This study opens the door to different avenues for future investigations. First, our pharmacological inhibition results can be confirmed using genetic deletion approaches to enhance the specificity of targeting Rho-ROCK and ITGB1. Interestingly, our preliminary data suggest that genetic deletion of ITGB1 using CRISPR-Cas9 seems to hinder astrocyte viability (data not shown). In addition, investigating the status of the embedded astrocytes in their reactive state would provide substantial information about how these astrocytes interact with their environment. Distinctions between reactive astrocyte states have been previously established into two subtypes: A1 and A2. While A1-activated astrocytes are conducive to a neurotoxic environment, through the expression of pro-inflammatory cytokines, A2 activation promotes a neural-protective state, allowing for survival and promotion of neurite regeneration.^57,58^ The specific reactive state of astrocytes could be indicative of the duality of the fibrotic scar and an explanation of why spontaneous regeneration fails when reversing astrogliosis. Finally, we can further modify the microenvironment, for instance by varying the ratios of collagen I to spinal cord ECM to mimic more acute or chronic stage of SCI. Following SCIs, aberrant ECM components like excess fibrillar collagen emerge in the lesion core.^59^ Our study shows that collagen I-reinforced spinal cord ECM fiber thickness can be successfully modified via cold- and warm-casting approaches and affect embedded astrocyte behavior. Varying the spinal cord to collagen ratios would allow more in-depth investigations of astrocyte reactivity via integrin engagement and mechanosensing. Future studies using this platform could explore the identification and further classification of astrocytes via additional customization of hydrogels to identify additional treatment groups and delineate astrocytes to a greater degree.

## 5 Conclusions

In this study, we have developed a platform that models the fibrotic scarring at the site of injury, causing an upregulation of key proteins in astrocytes, signifying astrogliosis. Overall, our work contributes to the current need for a better understanding of fibrotic scarring after SCI and highlights the importance of using 3D in vitro culture platforms with fine-tuned microstructural properties of ECM fibers more than their mere presence in understanding astrocyte behavior in the context of fibrotic scarring. Despite showing altered astrocyte phenotypes using immunofluorescence and pharmacological inhibitions, further investigation using genetic deletion of Rho-ROCK, fibronectin, or laminin may provide further insight into astrocyte roles in fibrotic scarring.

## Supporting Information Available

Individual statistical analysis of graphs shown in Figures 2–6 (PDF)
