# Supplementary material for: Development of Tissue-Engineered Model of Fibrotic Scarring after Spinal Cord Injury to Study Astrocyte Activation and Neurite Outgrowth In Vitro
Source: ACS Biomater Sci Eng. 2024 Sep 11;10(10):6545–57. doi: 10.1021/acsbiomaterials.4c01100 (PMC11480936; doi:10.1021/acsbiomaterials.4c01100)
Supplement: Supplementary file 1 — ab4c01100_si_001.pdf [file ab4c01100_si_001.pdf]

Supporting Information

Development of Tissue-Engineered Model of Fibrotic Scarring after Spinal Cord Injury to Study Astrocyte Activation and Neurite Outgrowth in vitro

Authors: Nikolas Ala-Kokko, Inha Baek, Younghye Song*

Affiliations: Department of Biomedical Engineering, University of Arkansas, Fayetteville, AR 72701

*Correspondence:

Younghye Song, Ph.D.

125 ENGR

790 W Dickson St

Fayetteville, AR 72701

Email: yhsong@uark.edu

Office: 479-575-5008

N-cadherin	Cold Control	Cold Anti-ITGB1	Cold Y-27632	Warm Control	Warm Anti-ITGB1	Warm Y-27632
Cold Control	N/A	****	****	****	****	****
Cold Anti-ITGB1	****	N/A	ns	***	ns	****
Cold Y-27632	****	ns	N/A	*	ns	****
Warm Control	****	***	*	N/A	ns	ns
Warm Anti-ITGB1	****	ns	ns	ns	N/A	***
Warm Y-27632	****	****	****	ns	***	N/A

Table S1: Statistical analysis and significant differences of immunofluorescence image analysis of N-cadherin shown in Figure 2C. *p<0.05, **p<0.01, ***p<0.001, ****p<0.0001. Two-way ANOVA, Tukey's multiple comparison test.

GFAP	Cold Control	Cold Anti-ITGB1	Cold Y-27632	Warm Control	Warm Anti-ITGB1	Warm Y-27632
Cold Control	N/A	****	***	****	***	****
Cold Anti-ITGB1	****	N/A	ns	ns	ns	***
Cold Y-27632	***	ns	N/A	ns	ns	****
Warm Control	****	ns	ns	N/A	ns	****
Warm Anti-ITGB1	***	ns	ns	ns	N/A	****

Table S2: Statistical analysis and significant differences of immunofluorescence image analysis of GFAP shown in Figure 2D. *p<0.05, **p<0.01, ***p<0.001, ****p<0.0001. Two-way ANOVA, Tukey's multiple comparison test.

α-SMA	Cold Control	Cold Anti-ITGB1	Cold Y-27632	Warm Control	Warm Anti-ITGB1	Warm Y-27632
Cold Control	N/A	ns	***	**	ns	ns
Cold Anti-ITGB1	ns	N/A	***	*	ns	ns
Cold Y-27632	***	***	N/A	ns	****	****
Warm Control	**	*	ns	N/A	***	***
Warm Anti-ITGB1	ns	ns	****	***	N/A	ns
Warm Y-27632	ns	ns	****	***	ns	N/A

Table S3: Statistical analysis and significant differences of immunofluorescence image analysis of α-SMA shown in Figure 2E. *p<0.05, **p<0.01, ***p<0.001, ****p<0.0001. Two-way ANOVA, Tukey's multiple comparison test.

Fibronectin	Cold Control	Cold Anti-ITGB1	Cold Y-27632	Warm Control	Warm Anti-ITGB1	Warm Y-27632
Cold Control	N/A	ns	ns	ns	ns	ns
Cold Anti-ITGB1	ns	N/A	ns	ns	ns	ns
Cold Y-27632	ns	ns	N/A	**	ns	ns
Warm Control	ns	ns	**	N/A	ns	ns
Warm Anti-ITGB1	ns	ns	ns	ns	N/A	ns
Warm Y-27632	ns	ns	ns	ns	ns	N/A

Table S4: Statistical analysis and significant differences of immunofluorescence image analysis of Fibronectin shown in Figure 3C. *p<0.05, **p<0.01, ***p<0.001, ****p<0.0001. Two-way ANOVA, Tukey's multiple comparison test.

Laminin	Cold Control	Cold Anti-ITGB1	Cold Y-27632	Warm Control	Warm Anti-ITGB1	Warm Y-27632
Cold Control	N/A	****	ns	***	****	**
Cold Anti-ITGB1	****	N/A	****	ns	ns	ns
Cold Y-27632	ns	****	N/A	**	****	**
Warm Control	***	ns	**	N/A	***	ns
Warm Anti-ITGB1	****	ns	****	***	N/A	***
Warm Y-27632	**	ns	**	ns	***	N/A

Table S5: Statistical analysis and significant differences of immunofluorescence image analysis of Laminin shown in Figure 4C. *p<0.05, **p<0.01, ***p<0.001, ****p<0.0001. Two-way ANOVA, Tukey's multiple comparison test.

YAP	Cold Control	Cold Anti-ITGB1	Cold Y-27632	Warm Control	Warm Anti-ITGB1	Warm Y-27632
Cold Control	N/A	****	ns	****	****	ns
Cold Anti-ITGB1	****	N/A	****	***	*	****
Cold Y-27632	ns	****	N/A	****	****	ns
Warm Control	****	***	****	N/A	****	****
Warm Anti-ITGB1	****	*	****	****	N/A	****
Warm Y-27632	ns	****	ns	****	****	N/A

Table S6: Statistical analysis and significant differences of immunofluorescence image analysis of nuclear: whole cell staining ratio of YAP shown in Figure 5C. *p<0.05, **p<0.01, ***p<0.001, ****p<0.0001. Two-way ANOVA, Tukey's multiple comparison test.

AUC - DRG	Cold Control	Cold Anti-ITGB1	Cold Y-27632	Warm Control	Warm Anti-ITGB1	Warm Y-27632
Cold Control	N/A	ns	*	ns	ns	*
Cold Anti-ITGB1	ns	N/A	*	ns	ns	*
Cold Y-27632	*	*	N/A	ns	ns	ns
Warm Control	ns	ns	ns	N/A	ns	*
Warm Anti-ITGB1	ns	ns	ns	ns	N/A	*
Warm Y-27632	*	*	ns	*	*	N/A

Table S7: Statistical analysis and significant differences of area under the curve for neurite outgrowth distance (μm) vs. outgrowth count shown in Figure 6D. *p<0.05, **p<0.01, ***p<0.001, ****p<0.0001. Two-way ANOVA, Tukey's multiple comparison test.

BDNF	Cold Control	Cold Anti-ITGB1	Cold Y-27632	Warm Control	Warm Anti-ITGB1	Warm Y-27632
Cold Control	N/A	***	*	ns	*	****
Cold Anti-ITGB1	***	N/A	****	*	****	****
Cold Y-27632	*	****	N/A	***	ns	****
Warm Control	ns	*	***	N/A	***	****
Warm Anti-ITGB1	*	****	ns	***	N/A	****
Warm Y-27632	****	****	****	****	****	N/A

Table S8: Statistical analysis and significant differences of BDNF secretion from 3D astrocyte cultures, graph shown in Figure 6E. *p<0.05, **p<0.01, ***p<0.001, ****p<0.0001. Two-way ANOVA, Tukey's multiple comparison test.
